# Development and validation of a nomogram prediction model for clinically significant prostate cancer combined with PI-RADS V2.1, MRI quantitative parameters and clinical indicators: a two-center study

**DOI:** 10.3389/fonc.2024.1467793

**Published:** 2024-11-22

**Authors:** Yunhui Chen, Long Yan, Jiang Xianmei, Gu Heyi, Xie Wei, Peng Chao, Dong Yanwen, Dong Shicun, Gao Chao, Yu Cui, Gu Peng, Liu Xiaodong, Tuo xiaoyu, Ling Bingbing, Ji Wenqing, Gao Kexian, Li Qingqing, Zheng Linglin, Zhu Yun, Zhao Lei, Hu Jihong, Zhao Wei, Yang Yaying, Hu Juan

**Affiliations:** ^1^ Medical Imaging Department, First Affiliated Hospital of Kunming Medical University, Kunming, Yunnan, China; ^2^ Medical Imaging Department, Gejiu People’s Hospital, Gejiu, Yunnan, China

**Keywords:** clinically significant prostate cancer, PI-RADS V2.1, apparent diffusion coefficient, prediction models, nomogram

## Abstract

**Objective:**

To develop and validate a multi-index nomogram prediction model for clinically significant prostate cancer(CSPCa) by combining the PI-RADS V2.1, quantitative magnetic resonance imaging (MRI) parameters and clinical indicators.

**Methods:**

A total of 1740 patients (75% in the derivation cohort and 25% in the internal validation cohort) and 342 patients (the external validation cohort) were retrospectively included in the MRI follow-up database of the First Affiliated Hospital of Kunming Medical University between January 2015 and April 2021,and Gejiu People’s Hospital between January 2020 and December 2022.Important predictors of CSPCa in MRI-related quantitative parameters, PSA-derived indicators, and clinical indicators, such as age, were screened. The Net Reclassification Improvement Index(NRI),Integrated Discrimination Improvement Index(IDI), and clinical decision curve analysis (DCA) were calculated to compare the performances of the different models. Receiver operating characteristic(ROC) curves and clinical calibration curves were used to analyze and compare diagnostic effects.

**Results:**

The AUC value, best cut-off value, specificity, sensitivity and accuracy of model 1(PI-RADS + PSAD) derivation cohort were 0.935, 0.304, 0.861, 0.895 and 0.872, respectively. The AUC values of the internal and external validation cohorts for model 1 were 0.956 and 0.955, respectively. The AUC value, best cut-off value, specificity, sensitivity and accuracy of model 2(PI-RADS +PSAD + ADCmean) derivation cohort were 0.939, 0.401, 0.895, 0.853 and 0.882, respectively. The AUC values of the internal and external validation cohorts for model 2 were 0.940 and 0.960,respectively. After adding the ADCmean to the model, the NRI(categorical), NRI(continuous) and IDI values were 0.0154, 0.3498 and 0.0222, respectively. There was no significant difference between the predicted probability and actual probability (p> 0.05).

**Conclusion:**

Models 1 and 2 had reliable, efficient and visual predictive value for CSPCa. The ADCmean is an important predictive indicator.

## 1 Introduction

Prostate cancer(PCa) is a common malignant tumor in men, and studies have shown that the incidence and mortality rates of this disease will continue to rise in China over the next 10 years (1). Prostate biopsy remains the gold standard for the diagnosis of PCa. However, because of its invasive nature and false-negative results, multiparametric magnetic resonance imaging (mpMRI) plays an increasingly important role in PCa detection (2),mpMRI examination before biopsy shows great value in the localization of lesion puncture, increasing the detection rate of cancer and reducing the number of puncture (3). To standardize and improve the detection of CSPCa, the Prostate Imaging Reporting and Data System V2.1 (PI-RADS V2. 1) was proposed in 2019 (4),which has better specificity, accuracy, and inter-reader consistency than PI-RADS V2.0 (5). However, there is room for further optimization in terms of diagnostic specificity and biopsy recommendations for lesions with a PI-RADS score of 3 (6), an original research revealed that among patients with negative MRI results and a meta-analysis revealed that among patients with negative MRI results and PI-RADS 3 lesions, incorporating PSAD into prostate biopsy decisions may aid in improving risk assessment and adjusting care (7, 8). Huang H et al. showed that in PI-RADS category 3 lesions, increasing the ADC value can enhance the predictive ability of CSPCa (9). The aforementioned research indicates that clinical indicators, such as prostate-specific antigen (PSA) and its derived indicators, as well as quantitative information, such as apparent diffusion coefficient(ADC) values from MRI, has the potential to enhance the application value of PI-RADS. Currently, most multi-index prediction models are based on PI-RADS V2.0, the joint application of quantitative information such as PSA and its derived indicators and ADC values is insufficient. Few studies have explored whether quantitative ADC value can improve PI-RADS and there is no research that simultaneously evaluates the predictive value of CSPCa by integrating PI -RADS, PSAD, and ADC values. Some models containing PSAD lack external data validation (10, 11). The aim of this study was to develop a visualized CSPCa prediction model based on PI-RADS V2.1, PSA and its derived indicators, ADC values, other quantitative parameters, and clinical indicators to perform internal and external data validation to improve the reliability of the prediction model and optimize detection efficiency to better guide clinical work.

## 2 Materials and methods

### 2.1 Study population

**Figure 1:**
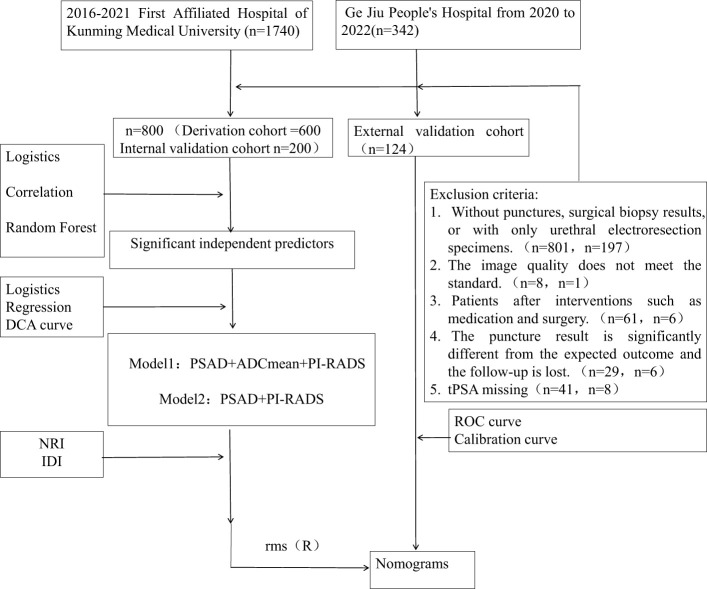
This diagram show the pathway of patient inclusion and exclusion, and the construction of CSPCa prediction model.

We retrospectively screened consecutive cases of prostate mpMRI examination for qualitative diagnosis from the prostate MRI follow-up database of the First Affiliated Hospital of Kunming Medical University (January 2015 to April 2021) and Gejiu People’s Hospital (January 2020 to December 2022) with the approval of the Ethics Committee. The interval between the MRI examination and biopsy or surgery should not exceed 3 months, and follow-up should be conducted for > 1 year. Based on the inclusion and exclusion criteria, 800 patients from the First Affiliated Hospital of Kunming Medical University and 124 patients from the Gejiu People’s Hospital were enrolled for model development and validation (**Figure 1**). All patients underwent MRI-perceived fusion hotspot biopsy combined with a systematic 12-needle transrectal ultrasound-guided prostate biopsy. According to the pathological nature, Gleason score, and 2014 International Society of Urological Pathology (ISUP) grading system, CSPCa was defined as an ISUP ≥ 2, a Gleason score ≥7, and/or prostate extracapsular invasion.

### 2.2 MRI scanning program

Cases in both centers’ databases were examined using 3.0T MRI equipment, with identical scanning parameters and consistent pre-examination preparation. Prostate mpMRI was performed at First Affiliated Hospital of Kunming Medical University using a 3.0T MR scanner (Achieva, Philips; Discovery MR W750, GE). The scanning instrument used for patients at Gejiu People’s Hospital was a 3.0T MRI combined with imaging UMR770. The patient was administered a low-residue diet and oral laxatives 24 hours before examination. On the day of examination, a glycerin enema was used for bowel preparation, and the bladder was moderately filled. The MRI scanning sequence included multi-planar T2 weighted imaging (T2WI), including a non-fat suppression sequence, T1 weighted imaging (T1WI), diffusion-weighted imaging (DWI), and dynamic contrast-enhanced (DCE) sequence. An abdominal phased-array coil is used as the receiving coil.T2WI: High-resolution T2WI in the axial, sagittal and coronal planes using at least the axial plane without a fat suppression. The scanning parameters for axial T2WI were as follows: repetition time TR 2900 ms; echo time TE 90 ms; thickness was 4 mm; interlayer spacing=0 mm; The field-of-view(FOV) was 26×26 cm(320×280 matrix); and excitation time=4. DWI: In the axial plane, a single-shot EPI sequence with b=0, 1000 and 1400 s/mm2; scanning parameters: TR 4000 ms, TE 70 ms, thickness=4 mm, interlayer spacing=0 mm, FOV was 26×26 cm(184×184 matrix), and excitation time=4. After fitting the DWI images at each b-value, we reconstructed and generated an apparent diffusion coefficient (ADC) map (3). dynamic contrast-enhanced(DCE): Utilize spatial sensitivity encoding technology, with scanning parameters: TR 3.15 ms, TE 1.6 ms, thickness=2 mm, number of layers=40, FOV was 40×40 cm(256×256 matrix). The contrast agent was gadolinium-doped diatrizoate injection or imported gadolinium-doped diatrizoate injection, injected through the elbow vein at a rate of 0.1 mmol/kg and a speed of2.5-3.0 ml/s. Before injecting the contrast agent, a plain scan was performed as a mask image, and scanning was started at the same time as the injection of the contrast agent with 20 consecutive scanning cycles, with a time resolution of 15 s. Dynamic contrast-enhanced scanning was performed using a T1 high-resolution isotropic volume acquisition sequence.

### 2.3 Statistical analysis

The same readers were used in both centers, and training and quality control were carried out prior to the scoring. Two experienced radiologists (with more than 5 years of experience in prostate MRI diagnosis) were responsible for performing PI-RADS V2.1 scoring. Patients with divergent scores were identified by an experienced deputy chief physician (with > 10 years’ experience in prostate MRI diagnosis) to determine the final score. Quantitative measurements were performed by an experienced radiologist (with more than five years of experience in prostate MRI diagnosis). Three-dimensional measurements of the prostate were performed on axial and sagittal T2WI according to the method recommended by PI-RADS V2.1. Prostate volume (PV) = (left-right diameter) × (anterior-posterior diameter) × (upper-lower diameter) × 0.52. PSA density (PSAD) = t-PSA/PV and f-PSA density = f- PSA/PV. ADC values were selected on the post-processing workstation to outline the lesion contour region of interest (ROI) on axial images, avoiding necrosis, hemorrhage and calcification areas.

All statistical analyses were conducted using R software version 4.2.2 for data analysis. Statistical significance was set at P<0.05. The differences between the three datasets and the comparison between CSPCa and non-CSPCa groups were analyzed using the Kruskal-Wallis test and Mann-Whitney U test for ADCmean, ADCmax and ADCmean, and ANOVA and t-test for the remaining variables. The “mice package” was used for multiple imputations to fill in missing values. Correlations between variables were analyzed using a heat-matrix plot. Univariate and multivariate logistic regression analyses were used to screen for the independent influencing factors. Random forest analysis (randomForest package) was used to further analyze the importance of each variable in CSPCa. Among the four most important variables in the random forest analysis, three categories of variables with significant or strong correlations with CSPCa were selected for model construction. To avoid collinearity and overfitting problems, indicators in the same category, such as PSA-derived or ADC-related parameters, were included as the most important parameters for modeling. Clinical decision curve analysis (DCA) was used to evaluate the net clinical benefits of the multiple modeling schemes. The net reclassification index (NRI) and integrated discrimination improvement (IDI) were used to evaluate ADC values to improve the model. The “rms package” was used to construct a nomogram and the training set was resampled using bootstrapping 1000 to construct a calibration curve. The “val.prob” function was used for calibration curve evaluation of the internal and external validation cohorts. The area under the receiver operating characteristic (ROC) curve was used to evaluate the degree of discrimination.

## 3 Results

### 3.1 Clinical characteristics of patients

**Figure 2:**
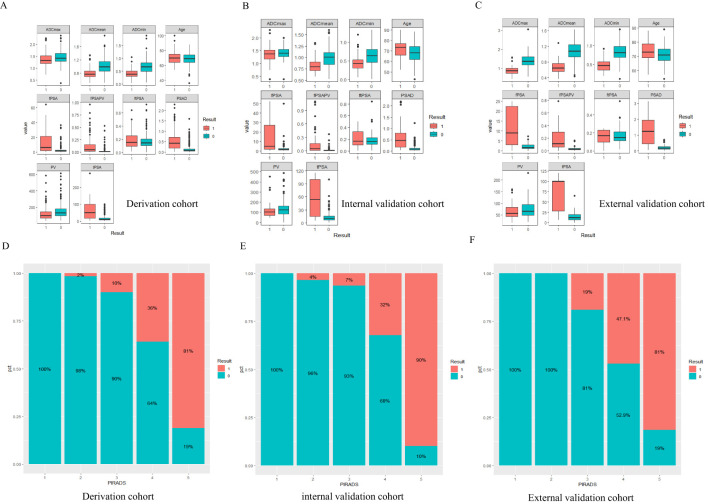
Result 0 is non-CSPCa, Result 1 is CSPCa, PIRADS=PI-RADS V2.1,fPSA=free prostate specific antigen, tPSA=total prostate specific antigen, ftPSA=fPSA/tPSA, PV=the volume of the prostate depend on MRI, fPSAPV=fPSA/PV, PSAD=tPSA/PV. **(A-C)** The boxplot of continuous variables in three datasets, Panels **(D-F)** CSPCa distribution of PI-RADS1-5 scores.

A total of 800 patients from First Affiliated Hospital of Kunming Medical University were randomly divided into derivation and internal validation cohorts in a ratio of 3:1. The derivation cohort included 258 patients with CSPCa and 542 patients with non-CSPCa, whereas the internal validation cohort included 66 patients with CSPCa and 124 patients with non-CSPCa. A total of 124 patients rom Gejiu People’s Hospital were included in the external validation cohort, comprising 34 patients with CSPCa and 90 patients with non-CSPCa. Comparing CSPCa and non-CSPCa, all indicators were significantly different (p < 0.05), except for ADCmax in the internal validation set (p = 0.242).There was no significant difference in the three groups of data comparison, except for f-PSA/PV, age, and PI-RADS scores, and the rest of the variables were not significantly different, and the details are shown in **Table 1** and **Figure 2**.

**Table 1:** Patients’ characteristics of the Derivation cohort and Internal validation cohort and External validation cohort.

Variable	Derivation cohort (600)				Internal validation cohort(200)				External validation cohort(124)				p
	Total1	CSPCa(n=191)	non-CSPCa (n=409)	P_1_	Total2	CSPCa (n=67)	non-CSPCa (n=133)	P_2_	Total3	CSPCa (n=34)	non-CSPCa (n=90)	P_3_	
PI-RADS score(Mean ± SD)	3.36 ± 1.28	4.68 ± 0.69	2.74 ± 1.00	<0.001	3.3 ± 1.31	4.69 ± 0.70	2.66 ± 0.98	<0.001	3.01 ± 1.33	4.53 ± 0.71	2.43 ± 1.02	<0.001	0.01
PI-RADS 1	19	0/19	19/19		11	0/11	11/11		11	0/11	32/11		
PI-RADS 2	181	4/181	177/181		56	2/56	54/56		48	0/48	48/48		
PI-RADS 3	151	15/151	136/151		46	3/46	43/46		21	4/21	17/21		
PI-RADS 4	64	23/64	41/64		28	9/28	19/28		17	8/17	9/17		
PI-RADS 5	185	150/185	35/185		59	53/59	6/59		27	22/27	5/27		
ADCmin×10^-3(Mean ± SD,s/mm2)	0.61 ± 0.24	0.40 ± 0.19	0.70 ± 0.24	<0.001	0.64 ± 0.27	0.45 ± 0.23	0.64 ± 0.26	<0.001	0.74 ± 0.28	0.48 ± 0.17	0.83 ± 0.25	<0.001	0.285
ADCmax×10^-3(Mean ± SD,s/mm2)	1.44 ± 0.23	1.37 ± 0.27	1.46 ± 0.25	<0.001	1.43 ± 0.25	1.37 ± 0.30	1.41 ± 0.24	0.242	1.25 ± 0.39	0.90 ± 0.25	1.38 ± 0.35	<0.001	0.194
ADCmean×10^-3 (Mean ± SD s/mm2)	0.96 ± 0.20	0.79 ± 0.14	1.04 ± 0.20	<0.001	1.00 ± 0.22	0.82 ± 0.16	1.00 ± 0.22	<0.001	0.97 ± 0.3	0.66± 0.17	1.09 ± 0.25	<0.001	0.227
Age(Mean ± SD,years)	69.1 ± 7.63	70.06 ± 7.67	68.72 ± 7.58	0.044	69.9 ± 8.54	71.93 ± 8.08	67.66 ± 8.43	<0.001	71.4 ± 6.9	73.71 ± 7.26	70.57 ± 6.71	0.025	0.009
t-PSA(Mean ± SD,ng/ml)	29.3 ± 33.1	57.14 ± 40.2	16.83 ± 21.89	<0.001	27.9 ± 31.2	55.00 ± 37.12	14.28 ± 14.9	<0.001	31.9 ± 33.8	135.57 ± 114.5	17.75 ± 16.3	<0.001	0.315
f-PSA (Mean ± SD, ng/ml)	6.3 ± 11.2	14.11 ± 16.3	2.70 ± 5.15	<0.001	7.33 ± 13.6	17.31 ± 19.71	2.58 ± 4.84	<0.001	6.22 ± 6.93	11.85 ± 9.76	2.64 ± 2.60	<0.001	0.178
f/t PSA	0.18 ± 0.11	0.20 ± 0.14	0.16 ± 0.09	<0.001	0.2 ± 0.14	0.22 ± 0.16	0.18 ± 0.11	0.017	0.17 ± 0.09	0.10 ± 0.06	0.17 ± 0.09	<0.001	0.493
f-PSA/PV(Mean ± SD, ng/ml2)	0.08 ± 0.10	0.20 ± 0.29	0.02 ± 0.05	<0.001	0.14 ± 0.34	0.18 ± 0.30	0.03 ± 0.09	<0.001	0.08 ± 0.13	0.20 ± 0.19	0.04 ± 0.04	<0.001	0.001
PV (Mean ± SD, cm^3)	73.2 ± 41.9	62.16 ± 77.1	78.47 ± 41.27	<0.001	62.3 ± 39.8	58.28.1 ± 68.7	71.61 ± 40.3	0.025	72.3 ± 35.7	69.21 ± 36.59	73.50 ± 35.5	0.553	0.069
PSAD (Mean ± SD,ng/ml2) (IQR)	0.49 ± 0.63	1.0 ± 0.8	0.25 ± 0.42	<0.001	0.54 ± 0.76	1.15 ± 0.52	0.24 ± 0.15	<0.001	0.53 ± 0.65	2.38 ± 2.26	0.25 ± 0.21	<0.001	0.695

### 3.2 Variable selection

**Figure 3:**
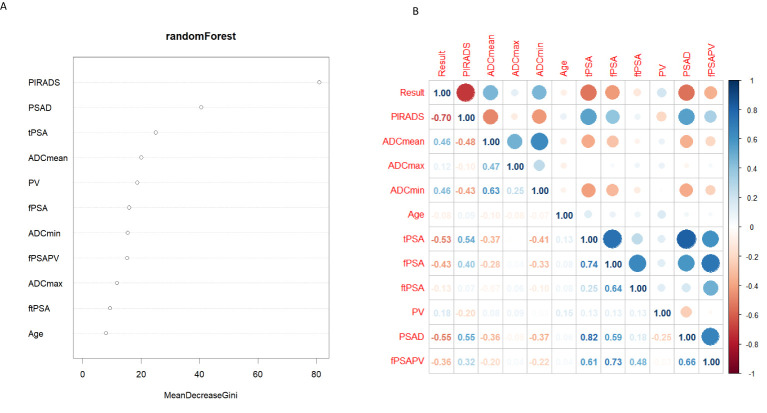
**(A)** Random Forest filter variables, Y-axis for variable names, X-axis for variable importance. **(B)** The correlation thermodynamic matrix graph was used to screen the variables. PIRADS, PI-RADS V2.1; fPSA, free prostate specific antigen; tPSA, total prostate specific antigen; ftPSA, f/tPSA; PV, the volume of the prostate depend on MRI; fPSAPV, fPSA/PV; PSAD, tPSA/PV.

The four most important parameters for random forest analysis (**Figure 3**) were ranked as follows: PI-RADS,PSAD, tPSA, and ADCmean. **Table 2** of the logistic regression analysis showed that all variables included in the univariate analysis had a P-value of less than 0.05. In multivariate analysis, the three parameters PI-RADS score, ADCmean, and PV had a P-value of less than 0.05. In the correlation heatmap (**Figure 3**), the top four variables with the highest correlation with CSPCa were PI-RADS (r=0.7), PSAD (r=0.55), tPSA (r=0.53), ADCmean, and ADCmin (r=0.46). Owing to the correlation between tPSA and PSAD, the importance of PSAD in the random forest analysis was higher than that of tPSA, ADCmin performed worse than ADCmean in the regression and random forest analyses. Finally, PI-RADS, PSAD and ADCmean were included to develop a nomogram.

**Table 2:** Logistic regression.

Variable	Single factor logistic regression			Multivariate logistic regression		
	OR	95%CI	P	OR	95%CI	P
PI-RADS	6.350	4.834~8.342	<0.001	3.494	2.600~4.694	<0.001
ADCmin	0.001	0.001~0.004	<0.001	0.205	0.037~1.121	0.067
ADCmax	0.259	0.121~0.553	<0.001	1.888	0.479~7.446	0.364
ADCmean	0.000	0.001~0.001	<0.001	0.073	0.007~0.787	0.031
Age	1.024	1.001~1.047	0.045	1.011	0.973~1.049	0.580
t-PSA	1.041	1.034~1.049	<0.001	1.023	0.952~1.019	0.088
f-PSA	1.141	1.101~1.183	<0.001	1.059	0.966~1.106	0.222
f/t PSA	21.311	4.112~110.43	<0.001	6.071	0.015~140.55	0.279
f-PSA/PV	8278.3	3806.9~18010.0	<0.001	0.212	0.002~6.941	0.308
PSAD	305.65	102.06~915.36	<0.001	0.702	0.212~2.327	0.563
PV	0.994	0.991~0.997	<0.001	0.977	0.965~0.989	0.001

### 3.3 Constructing nomograms and verifying internal and external data

**Figure 4:**
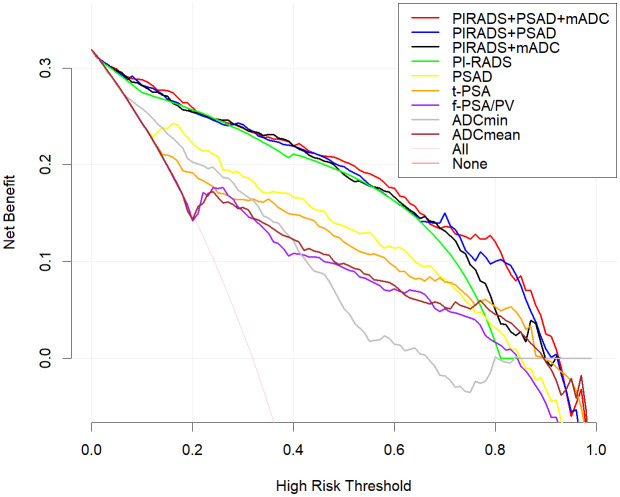
Clinical decision curve analysis, the Y axis represents the net benefit and the X axis represents the risk threshold.Within the threshold probability range of decision curve analysis, the PI-RADS + PSAD + ADCmean model provided best clinical.benefit. Compared with ADCmin and, ADCmean is more suitable for clinical decision. Of all PSA-derived parameters, PSAD had the best clinical benefit, but less than the PI-RADS score.

**Figure 5:**
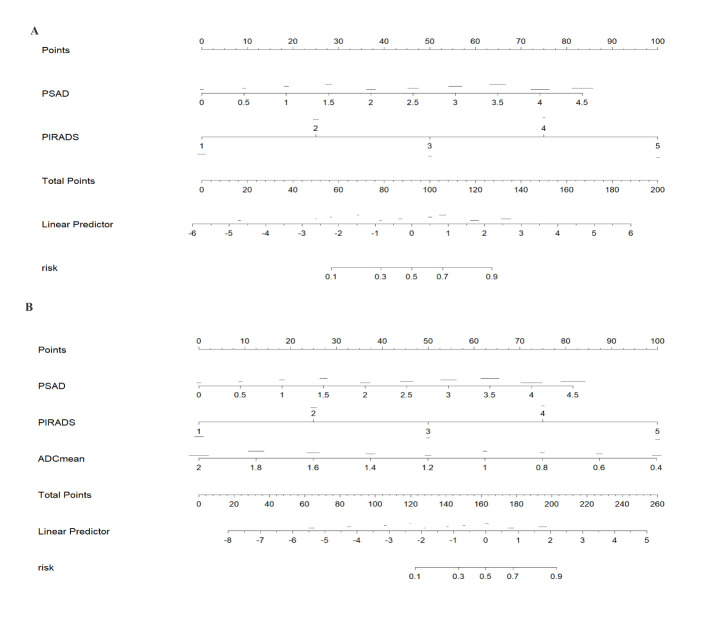
The nomograms of models 1 and 2 was drawn in rms package of R. Model 1 constructs a nomogram for predicting clinically significant prostate cancer (CSPCa) using PIRADS (PI-RADS 2.1) and PSAD (tPSA density = tPSA/prostate volume PV). Model 2 adds ADCmean on the basis of Model 1. According to the above variables, the risk of CSPCa can be determined. Model 1 **(A)** constructs a nomogram for predicting clinically significant prostate cancer (CSPCa) using PIRADS (PI-RADS 2.1) and PSAD (tPSA density = tPSA/prostate volume PV). Model 2 **(B)** adds ADCmean on the basis of Model 1. According to the above variables, the risk of CSPCa can be determined.

**Figure 6:**
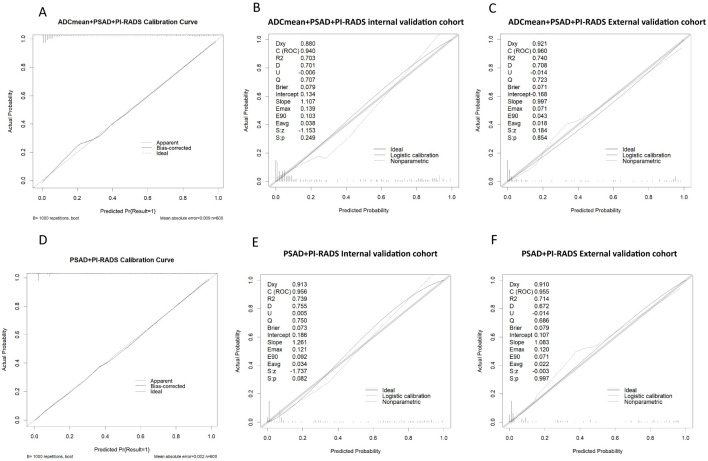
**(A-C)** model 2 Calibration curve in derivation cohort, internal validation cohort, external validation cohort. **(D-F)** model 1 Calibration curve in derivation cohort, internal validation cohort, external validation cohort.

Among the various modeling schemes, the clinical decision curve (**Figure 4**) showed that models 1(PSAD + PI-RADS) and 2 (PSAD + PI-RADS + ADCmean) had the highest clinical benefits. The constructed nomogram was shown in **Figure 5**. Comparing Models 1 and 2, NRI(categorical)=0.0154,P-value=0.16252, NRI(continuous)=0.3498, P-value=0.00005, IDI=0.0222, and P-value=0.00029 are detailed in **Table 3**. The AUC value, best cutoff value, specificity, sensitivity, and accuracy of the model 1 derivation cohort were 0.935, 0.304, 0.861, 0.895, and 0.872, respectively. The AUC values of the internal and external validation cohorts for model 1 were 0.956 and 0.955, respectively. The AUC value, best cutoff value, specificity, sensitivity, and accuracy of the model 2 derivation cohort were 0.939, 0.401, 0.895, 0.853, and 0.882, respectively. The AUC values of the internal and external validation cohorts for model 2 were 0.940 and 0.960, respectively. The internal and external validation of the calibration curve are shown in **Figure 6**. The clinical calibration curve showed no significant difference between the predicted and actual probabilities of the two models (p > 0.05) (**Figure 6**).

**Table 3:** Reclassification table.

Outcome: absent				
		Updated Model=ADCmean+PSAD+PIRADS		
Initial Model= PSAD+PIRADS		[0,0.5]	[0.5,1]	reclassified
	[0,0.5]	372	0	0
	[0.5,1]	2	35	5
Outcome: present				
		Updated Model=ADCmean+PSAD+PIRADS		
Initial Model= PSAD+PIRADS		[0,0.5]	[0.5,1]	reclassified
	[0,0.5]	33	3	8
	[0.5,1]	1	154	1
Combined Data				
		Updated Model=ADCmean+PSAD+PIRADS		
Initial Model= PSAD+PIRADS		[0.5,1]	[0,0.5]	reclassified
	[0,0.5]	405	3	1
	[0.5,1]	3	189	2

A cutoff of 0.5 was used to calculate IDI, NRI:

NRI(Categorical) [95% CI]: 0.0154 [-0.0062 - 0.0369]; *P-value*: 0.16252.

NRI(Continuous) [95% CI]: 0.3498 [0.1815 - 0.5182]; *P*-*value:* 0.00005.

IDI [95% CI]: 0.0222 [0.0102 - 0.0343]; *P*-*value:* 0.00029.

## 4 Discussion

In this study, we conducted rigorous variable screening, model development, and validation of clinical indicators, such as PI-RADS V2.1, MRI quantitative parameters, PSA and its derived indicators and clinical indicators using multiple methods. The results showed that modeling with PI- RADS, PSAD and ADCmean had the best clinical benefits, with a correlation coefficient of 0.53 for tPSA and 0.55 for PSAD, which are the two most important PSA-derived indicators. Some prospective cohort studies that received PSA testing suggested that the combined AUC values of tPSA and f/tPSA were significantly lower than those of PSAD, and using PSAD could help avoid unnecessary biopsies (12, 13). PSAD appears to be a useful marker that can stratify the risk of CSPCa in a complementary manner to prostate mp-MRI (14). DENIFFEL D et al. showed that the number of unnecessary prostate biopsies in men with positive MRI may be safely reduced by using a prostate- specific antigen density-based strategy (15), which is consistent with our findings. In our study, ADCmean and ADCmin had the same correlation coefficient (0.46), indicating moderate correlation. The DCA curve showed that the clinical decision-making benefit of ADCmean was higher than that of ADCmin, making ADCmean the most valuable reference indicator among the ADC-related values measured by contouring. Overall, the importance of each index was in the following order: PI-RADS>PSAD>ADCmean. Tezcan et al. also showed that PI-RADS V2.1 was a stronger predictor of CSPCa than PSAD (16). In this study, when the most important PI-RADS was used to predict CSPCa, 10%, 7%, and 19% of CSPCa cases were detected in the training, internal validation, and external validation sets, respectively, when the PI-RADS score was 3. Two other studies showed that the CSPCa detection rates were 16% and 12% when the PI-RADS score was 3. A systematic review showed that the detection rates of prostate cancer using PI-RADS 1-5 were 6%, 9%, 16%, 59%, and 85%, respectively (17, 18). Compared with the above studies, our score detected fewer positive patients in scores 1 and 2, and the other categories had similar accuracy (**Figure 2**). The PI-RADS alone predicted an AUC value of 0.910, and the specificity under the optimal cutoff value was 0.814, which was lower than that of the joint model (0.895). The AUC values of joint modeling models 1 and 2 reached 0.935 and 0.939, respectively, indicating that multiple factors can jointly improve the accuracy of disease diagnosis. The AUC values in both internal and external validations of the two joint schemes were all greater than 0.930, indicating a good generalization ability.

**Figure 7:**
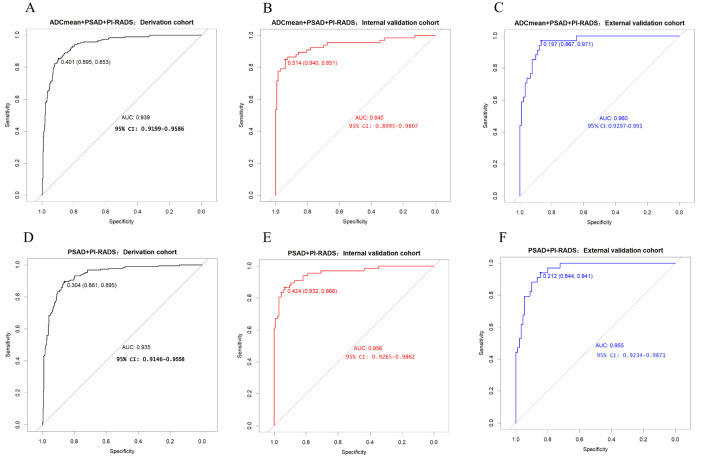
**(A-C)** model 2 receiver operating characteristic curve in derivation cohort, internal validation cohort, external validation cohort. **(D-F)** model 1 receiver operating characteristic curve in derivation cohort, internal validation cohort, external validation cohort.

Nomograms are increasingly being applied to clinical multi-index joint decision-making for various diseases, owing to their simplicity and strong visualization. Our study demonstrated that the performance of the nomogram prediction model for CSPCa was high (**Figure 7**). RODRIGUEZ et al. (19) and WANG L et al. (9) used PI-RADS V2 and PSAD nomograms for PCa diagnosis, with AUC values of 0.803 and 0.95, respectively. Ma Z et al. (20) studied the prediction of CSPCa based on PI- RADS V2.1 + PSAD + Age nomograms, with a combined model AUC of 0.938 (95% CI, 0.922-0.955) and a validation set AUC value of 0.914 (95% CI, 0.873-0.955). The above studies included PSAD and PI-RADS scores in variable screening for modeling. The performance of the PI-RADS V2 prediction model varies greatly, and MA Z et al. and our study were based on PI-RADS V2.1, with CSPCa as the predictive outcome. The results show high AUC values and external validation performance, which may indicate that the Version 2.1 has a more stable prediction performance. As age was not a significant predictor of CSPCa in our study, it was not included in the modeling process. Meanwhile, although this study did not limit the use of the model to specific patients with a PI-RADS score of 3 or a negative first puncture (3, 8, 9), in the external validation set, for example, 20 out of 124 patients had a PI-RADS score of 3, of which 4 cases were diagnosed with CSPCa, and a total of 4 cases of CSPCa were missed by the PI-RADS score on a scale of 1-3 as negative and 4-5 as positive; both models were able to avoid the missed diagnosis of 2 of these cases. A total of 3 patients with negative first puncture in 124 patients underwent a second puncture, which confirmed the diagnosis of 1 case of CSPCa, and both models accurately diagnosed 1 case of positivity and 2 cases of negativity. This suggests that both models in this study, can cover the diagnosis of a specific patient well and be more widely used for the generalized diagnosis of PCa.

In this study, two indices, IDI and NRI, were used to evaluate model differences after the addition of ADCmean: NRI(categorical)=0.0154, NRI(continuous)=0.3498, and IDI=0.0222, this showed that the ADCmean value had a positive effect on the model. Liu et al. (21) showed that the combination of ADC values with PI-RADS V2.1, was not significantly better than PI-RADS V2.1 alone for all patients with PI-RADS 1-5, but in PI-RADS 3-4 lesions, the combined AUC value of ADC and PI- RADS V2.1 scores was 0.756 (95% confidence interval, 0.646-0.846), which was significantly higher than 0.631 (95% confidence interval, 0.514-0.738) for PI-RADS V2.1 alone (P=0.047). **Figure 4** showed that within a risk threshold of greater than 0.6, the clinical benefit of PI-RADS+ADCmean was greater than that of PI-RADS alone. TAVAKOLI A A study found that ADC values less than or equal to 0.90 × 10−3 mm2/sec supported upgrading of ADC PI-RADS 3 and ADC PI-RADS 4 lesions (22). Quantitative ADC measurement may be more important for risk stratification than current methods in future versions of PI-RADS (23). We believe that the ADCmean value is an important CSPCa prediction parameter and that the internal and external validation of the model containing this parameter has high AUC values. The calibration curve showed that the model prediction probability was roughly similar to the actual probability, indicating that the measurement of ADC values in different regions and devices to identify CSPCa still had a high application value and was an important parameter that was not ignored, providing more prediction accuracy. In view of the increased workload of ADC value measurement, which may hinder the application of the model, we constructed two columns of Model 1 and Model 2 for different clinical application scenarios, considering the different emphases on the need for accuracy and convenience (**Figure 5**).

This study had several advantages in terms of variable selection. Adding the random forest method reduced the impact of collinearity in the logistic regression and fully evaluated the clinical benefits of different modeling schemes using decision curves, ensuring that the best indicators and optimal schemes were not missed to the greatest extent. Similar to other retrospective studies, our study had a risk of selection bias. As the two centers were located in the same region, the proportion of patients suspected of PCa due to elevated PSA may be relatively lower, and the proportion of patients suspected of PCa due to urologic symptoms such as hematuria and urinary tract obstruction may be relatively higher compared with other more developed regions, and as both hospitals were regional centers for PCa treatment, the sample may have had a relatively high proportion of CSPCa and an higher proportion of advanced cancer, which may have resulted in a higher accuracy of the PI-RADS score and a higher AUC value of the model. And there may be differences in the performance of the PI-RADS scores between different centers and radiologists with different experience in reading films. Compared with similar studies, our diagnostic model has a higher AUC value, which may pose a certain risk of overfitting. In conclusion, although the present study has been statistically designed to ensure the objectivity and stability of the model as much as possible, large-scale applications may require more prospective datasets from more centers for validation due to the risks of selection bias and overfitting. Owing to the differences in different subzones of the prostate, especially in the peripheral and transverse zones, the value of prostate ADCmean zoning measurements in improving the model deserves further exploration. And there is possibility of further improvements in model performance regarding application of blood-based protein biomarkers (e.g., PHI), urine-based gene expression assays (e.g., PCA3), and other biomarkers.

## 5 Conclusion

The PI-RADS+PSAD+ADCmean and PI-RADS+PSAD nomogram models have high predictive value and good generalization ability, providing valuable evidence for clinical diagnosis and guiding the development of individualized diagnostic and treatment plans to avoid unnecessary punctures. PI- RADS+PSAD nomogram mode l is more convenient and has better detection efficiency, whereas ADCmean is an important predictive indicator that can improve the diagnostic performance of mode l to a certain extent.

## Data Availability

The corresponding author can provide the reader with the raw data. Requests to access these datasets should be directed to HJ, hujuan_1111@163.com.
